# Playing the role of a ‘boundary organisation’: getting smarter with networking

**DOI:** 10.1186/1478-4505-9-S1-S11

**Published:** 2011-06-16

**Authors:** Scott Drimie, Tim Quinlan

**Affiliations:** 1 International Food Policy Research Institute (IFPRI; 2 Health Economics and HIV/AIDS Research Division (HEARD), University of KwaZulu-Natal

## Abstract

**Background:**

This paper discusses the practices of organisations that cross the boundary between research and politics, to promote evidence-based policies and programmes.

**Methods:**

It uses the experience of a network of organisations in Africa to describe the methodology, challenges and successes of efforts to promote utilisation of research on the inter-connections between HIV/AIDS, food security and nutrition in South Africa. It emphasises that crossing the boundary between science and politics can be done systematically and is inevitable for any attempt that seeks influence policy making.

**Results:**

The paper reveals the complexity of the research-policy making interface and identifies key lessons for the practice of networking and engaging policy and decision-makers.

**Conclusion:**

The concept of boundary organisation is a helpful means to understand the methodological underpinnings of efforts to get research into policy and practice and to understand the ‘messy’ process of doing so.

## Introduction

This paper examines researchers’ practices to promote the utilisation of research. We use the concept of “boundary organisations”, signifying organisations that cross the boundary between science and politics and draw on the interests and knowledge of agencies on both sides to facilitate evidence-based and socially beneficial policies and programmes [1]. The term, to our knowledge, gained currency in the USA in the 1990s following efforts to combine climate research and weather forecasting, which led to the establishment of organisations that could speak to, and work with different agencies for the purpose of ensuring reliable seasonal climate forecasts [2,3]. The term may be relatively new but the concept has a longer heritage; for example, the linking of agricultural research to agricultural extension services to enhance national farm production in the USA early in the 20^th^ century [4]. Furthermore, application of the concept is well established internationally. For instance, agencies such as the UNAIDS plays this role; illustrated by its international consultative meetings on large public health issues that include researchers, politicians, activists and NGOs. Their role in southern Africa has been to facilitate research on behalf of organisations such as the Southern African Network of People Living with HIV to inform the messages imparted to their members. Likewise, the World Health Organisation (WHO) increasingly plays this role as reflected in its public profile during early phases of the ‘swine flu’ epidemic and its support for a global symposium in 2010 on health systems research.

Our contention is that organisations which cross the boundary between science and politics, consciously and systematically, play a deliberate role to facilitate evidence-based and socially beneficial policies and programmes and they draw on a range of strategies to achieve this. Networking may be one of these strategies but networks alone may not achieve the intended outcomes. Other strategies are needed such as building the confidence and capacity of different individuals and agencies over time such that they can actively make changes in their work. Similarly various communications strategies are needed to draw on the interests and knowledge of agencies on both sides. Innovative ways are also needed to ensure that bridges are built and maintained between different sectors.

The focus here is on the collaboration of the Regional Network on AIDS, Livelihoods and Food security (RENEWAL), a programme that engages government officials on research that could and should inform policies and programmes to address population vulnerability (food insecurity and HIV/AIDS) in South Africa. The paper examines the experiences of building networks and facilitating interactions with ‘champions’ in government departments to encourage greater use of research evidence in departmental development programmes; the underlying dynamics of boundary crossing; and the experience of getting research into policy or practice.

The purpose is to draw out lessons from these processes with regard to engaging and influencing policy/decision-makers to good effect.

## Background

RENEWAL is a regional network-of-networks. Active in Kenya, Malawi, South Africa, Uganda, and Zambia, RENEWAL comprises national and regional networks of food and nutrition-focused organisations and HIV/AIDS and public health organisations. These networks are based on previous financing of research studies in those countries by RENEWAL (a total of 17 studies between 2001 and 2007) and the development of conceptual frameworks, methodologies and indicators relating to HIV and vulnerability (http://www.ifpri.org/renewal). RENEWAL’s agenda is to enhance understanding of the intersections and links between HIV/AIDS and food and nutrition security (the ‘HIV-Hunger nexus’), and to facilitate interventions.

In order to perform as a boundary organisation RENEWAL has systematically set out to do more than be a network. Within each country of operation, locally-prioritised action research, capacity strengthening, and communications have enabled the networking approach to build relationships and trust that have allowed decision makers to access evidence for policy dialogue. Furthermore, in each country, RENEWAL has facilitated the establishment of ‘National Advisory Panels’ (NAPs) consisting of senior government officials, NGOs and academics with interests in the type of research being supported in that country. The NAPs take different forms but, they are channels to review and spread information from the research projects to relevant other officials and decision-making fora within the country. They also guide RENEWAL on which facets of current research projects are of interest to policy makers in that country as well as research topics which are likely to interest policy makers in the near and medium-term future. The operational ethos is that the process of developing networks is both a means and an end. Networking is necessary to create channels for identifying socio-economic challenges, for determining appropriate investigation, and for sustaining communication and information flows during research and during discussions on the potential and actual uses of research.

This paper assesses the concept of a boundary organisation in relation to RENEWAL’s agenda outlined above and the theoretical and methodological literature on policy making processes and the research-policy interface. This means of analysis allows for key lessons to emerge in terms of where and how RENEWAL has had an impact on policy making processes in South Africa and, with regard to methodology, on the role of networking within efforts to get research into policy and practice.

## Methods

The following section is divided into two parts. The first introduces the process and approach of the RENEWAL network in order to present operational challenges and successes in promoting utilisation of research on the inter-connections between HIV/AIDS, food security and nutrition in South Africa. The second part conceptualises boundary organisations in terms of using appropriate literature to give substance to the analysis in the subsequent sections. The method section as a whole emphasises that crossing the boundary between science and politics can be done systematically and is inevitable for any attempt that seeks influence policy making.

RENEWAL South Africa is facilitated by the Health Economics and HIV/AIDS Research Division (HEARD) at the University of KwaZulu-Natal in consultation with the International Food Policy Research Institute (IFPRI). RENEWAL established a NAP consisting of representatives of government departments engaged in food security and HIV/AIDS policy-making, HEARD and the Medical Research Council. This was done in view of these issues being themes within the projects that RENEWAL supported in the country. For South Africa, the NAP was conceived as a ’light’ panel in the sense of having few people and to be no more than a loose affiliation of agencies who would engage on these issues in a spirit of finding common ground. This was to avoid over-burdening hard-pressed officials and, as importantly, to accommodate political sensitivities associated with HIV/AIDS, poverty and nutrition at the time. In brief, there was an attitude described as “AIDS denialism at the highest level” of government [5]. In practice the NAP attracted middle to senior level officials whose professional responsibilities were to oversee programmes dealing with vulnerability and food insecurity.

The primary objectives of RENEWAL South Africa were to reduce gaps in knowledge on the relationship of livelihoods and food security with HIV and AIDS. Consequently, there were practical expressions of this agenda. For example, RENEWAL supported several research projects that adopted an action-research methodology while HEARD conveyed the agenda in terms of actively influencing interventions to be based on sound research and, conversely, to undertake research that served the design of effective strategies and policies.

HEARD defines itself overtly as a ‘learning organisation’ which has been expressed via various practical initiatives over time; for example, internally, through articulation of its organisational values, elaboration of mentoring mechanisms and reflexive review of networking and its networks; and externally through collaborative research projects that emphasised mutual capacity building and partnerships with other African research organisations.

Although this approach of setting up a “boundary organisation” was based on a practical response to “bridging the divide” in South Africa, experiences of boundary organisations elsewhere provide a useful way to interrogate the successes and challenges of RENEWAL. The second part of the methods section sets up key arguments based on “boundary organisation” literature in order to provide detailed results from the RENEWAL experience.

The Overseas Development Institute (ODI) acknowledges that many policy making processes are weakly informed by research-based evidence [7,8]. Policy-makers tend to be influenced by their own values, experience and judgement, lobbyists and pressure groups, and pragmatism. For example, at an ODI Conference, a former decision-maker in the UK argued that researchers and policy-makers have a completely different understanding of what constitutes good evidence:

“Researchers only consider their results to be reliable if they are proven scientifically and underpinned by theory, and are reluctant to say anything until it is. Even then, they tend to wrap their results up in caveats and qualifications. Policy-makers will take more or less anything that can help them to make a decision that seems reasonable, has a clear message and is available at the right time” [9].

Accordingly, researchers need to be cognisant of these factors. Investigating and working with the strategic demarcation between political and scientific tasks has been called “boundary work” [1]. Boundary organisations involve the participation of actors from both sides of the boundary, as well as individuals and organisations who play a mediating role but they have distinct lines of accountability to each. In other words, boundary organisations perform tasks that are useful to both sides, but have a distinctive role that would be difficult or impossible for organisations in either community to play.

The concept of boundary organisations helps practitioners and researchers to consider how blurring the boundaries between science and politics, rather than maintaining their separation (which is often advocated and practiced), can lead to more productive decision-making. This is a field in which there is a growing body of related investigations which, together, affirms the creation of new knowledge and innovative ideas that come from sharing diverse perspectives. To illustrate, this ranges from the field of environmental research and management, including impacts on health [10-14], to explorations of the ‘learning ethos’ within the scientific community [15,16], development sector [17,18] and NGO communities [19], to methodological exegeses by scientists [20,21 and trans-disciplinary research [22,23].

A successful boundary organisation serves two sets of agents and is itself the agency which bridges the boundary between them. To fulfil this role, a boundary organisation must have credibility on both sides of the boundary which, in the first instance, is achieved on the basis of engagement and inclusion of interested parties. Thereafter, credibility is maintained by the boundary organisation being acknowledged as an arbiter of the quality and utility of relevant research and its facilitation of an effective flow of information. Adopting a flexible, adaptive approach is essential for a boundary organisation; hence, a ‘network’ presents itself as one appropriate form for a boundary organisation.

Networks (and partnerships) are a more common term than boundary organisation for the work of agencies that promote information flow, knowledge sharing and communication between actors who might otherwise not be in touch [24]. However, as we indicated in the background section, networks entail a range of strategies and actions to build and maintain. It is the dynamic and organic nature of networking and networks, respectively, which pose challenges for ensuring that they function to good effect; hence, as we have indicated above, the need to appreciate their methodological underpinnings. In turn, it is this understanding which enables critical assessment of the value and utility of any network in a particular context as we discuss below.

The work undertaken by RENEWAL in the period 2006 to 2009 is analysed below using the concepts and approaches laid out. In particular five main areas of work will be investigated. Firstly an analysis of how RENEWAL attempted to get stakeholders to understand partnerships drawing on two research projects in South Africa. Secondly, the politics and practice of boundary crossing is explored. Thirdly, the need for making space for opportunities for action is assessed. Fourthly, the extractive nature of science is analysed, as experienced by RENEWAL. Finally, experiences of capacity strengthening are reviewed.

The data described in the article were largely collected by the authors as “insiders” to the RENEWAL process. Both played key roles in establishing and facilitating the network, overseeing the research components and working with different stakeholders to set up a “research-policy” interface. A review was undertaken of project documentation from this period including three annual donor reports, various outputs from four research projects conducted in South Africa including final research reports and formal publications, and email correspondence between members of the network. Some discussions were held with key members of the advisory group established to facilitate the interface mentioned above. These discussions were held in 2009 as part of a review process that fed into the proposal for a subsequent phase of RENEWAL. Records of these discussions were reviewed and where necessary follow up questions provided to the respondents.

## Results

Here, we discuss RENEWAL’s operational successes and challenges in South Africa between 2006 and 2009. The common denominator of the successes and challenges is the articulation of trust which underpins a set of mutual interests and support, which has long been recognised as a key factor in the operation of networks and partnerships [25-28] and which a boundary organisation seeks to hold amongst a range of organisations. This is in distinction to the instrumental purpose of networking and formal partnership contracts.

### Getting ‘stakeholders’ to understand ‘partnerships’

A recurring challenge has been to manage the ‘messiness’ of engaging with different organisations and the political vagaries of changing political conditions for researchers and policy/decision-makers. Frequently, the challenge has been to convince principal agencies (funders, research management committees, and partner organisations), to appreciate that ‘stakeholder consultation’ prior to research and dissemination of results via ‘feedback workshops’ are principles (and activities with various ramifications), which need to be applied throughout the research process. They are not discrete events that once done can be ticked off as ‘best practice’.

To illustrate, one RENEWAL-funded project in South Africa that addressed the challenges of improving rural livelihoods, health and nutritional security, obtained support from the Department of Health officials and clinics in the study locality at the start and during the course of the research. However, once the opportunities for collaborative intervention by different government agencies, NGOs and community organisations had been identified and agreed in principle by all, the process faltered due to lack of engagement by health officials with the opportunities to modify health care procedures [29].

Another project that explored the interactions between hunger, HIV and TB, culminated in a workshop in an urban settlement where the results were shared with research participants, residents, local officials and NGOs. The research results showed widespread co-infection and stimulated vocal criticisms amongst the participants and residents about the lack of effective health service support (e.g. slow time for diagnosis, inexperienced doctors). Subsequently, one workshop resolution was that the project had a responsibility to articulate and demand for better services. Furthermore, community representatives felt strongly that all should march to the offices and homes of local politicians to highlight their concerns. However, some of the researchers raised concerns that the study was being used for a larger political agenda which went beyond the actual focus and results of the study. Nonetheless there was a realisation amongst the researchers that their research had the power to evoke responses much greater than originally expected. In short, this was a case of researchers coming face to face with the difference between the dissemination and the utilisation of research.

Both cases provide a salutary lesson for researchers. They highlight the fact that the position of a researcher is frequently one without power to cause material change. Both cases reveal the how evidence alone, generated by researchers is insufficient to bring change. Other agencies have that capacity, for instance advocacy or civil society organisations. Hence the instrumental value of research and the credibility of the researcher lies ironically in their ‘powerlessness’: in the perceived ‘objectivity’ and ’neutrality’ of the researcher (and his/her attention to experiences of people) amongst other parties with different interests in a project. The capacity of the researcher to influence change lies in working with other parties that do have power, in ways that support them to use their power constructively.

### The politics and practice of boundary crossing

Another challenge for RENEWAL was dealing with the politics within science. RENEWAL and HEARD’s research strategies endorsed crossing disciplinary boundaries. Inter-disciplinary research can be initiated with relative ease; collaboration begins by working with like-minded individuals and organisations. However, as researchers work together, particularly in arenas as complex as the HIV-Hunger nexus, challenging questions emerge with regard to what is credible and salient knowledge; how does the means of generating this knowledge give it validity; and what type of knowledge should take precedence over others? In other words, researchers have to confront challenges both ways - to the empiricist foundations of science and, from those foundations, to constructivist approaches [30].

The RENEWAL strategy has been to adopt an ‘in-reach’ as opposed to an ‘outreach’ approach when starting projects. ‘Outreach’ implicitly emphasises difference and boundaries between different agencies. ‘In-reach’ implicitly acknowledges common concerns of different agencies and the scope for reaching in to the source of those concerns. The actions to that end were not unusual. Establishing and engaging with a National Advisory Panel has been a means to ensure ongoing consultation with stakeholders, to identify research priorities and changing political interests and perspectives on them. Means to strengthen consideration of the complexity of the HIV-Hunger nexus include regular interaction of RENEWAL’s co-ordinators with each other and, in each country, with all members of research teams, between senior team members from different institutions with each other in the case of cross-organisational studies, and between the principals of RENEWAL projects in different countries. These interactions are designed to ensure cross-site comparability in the design of projects and policy relevance, and they are a conduit for ongoing advocacy. With regard to advocacy, the various activities are a rich source of ideas and information for development and elaboration of communications and marketing strategies and outputs. In other words, ‘research communication’ is not simply dissemination of research results but can incorporate activities ranging from capacity building in the form of mentoring on writing for different media and training to write ‘issues briefs’, to using different media for different purposes as a project progresses, and ultimately, to packaging and disseminating the form of the research as well as the results in imaginative ways.

The ‘in-reach’ agenda is to maximise ownership, sustainability and influence of the work from the outset, and to ensure national, regional and international relevance of the results. In essence, it encourages the flow of knowledge in many directions thereby blurring further the distinctions between ‘pure’ and ‘applied’ research [6]. Nonetheless, there have been practical challenges to ensure this flow. The NAP, for example, is a means for practitioners and policy-makers to become more than recipients of scientific knowledge and to help configure research on the HIV-Hunger nexus. However, research budgets are predetermined and controlled by the IFPRI in Washington which has led to tensions about ‘who sets the (budgeted) agenda’. As the NAPs began to fulfil their roles, there were many demands on the RENEWAL budget. Funding commitments needed to be modified yet reconciled with donor contracts and the original proposal developed by IFPRI. In sum, creating the links can take time to bear fruit but the costs cannot always be predicted precisely.

### Making space for opportunities to be taken

RENEWAL South Africa has benefited from the active facilitation of discussions amongst researchers and government officials by HEARD, the Centre for the Study of AIDS at the University of Pretoria (CSA) and the Nutrition Directorate in the National Department of Health. These discussions have stimulated ideas for projects that support government interests. There is work-in-progress with the Africa Centre for Food Security at the University of KwaZulu-Natal on building the capacity of young researchers and government officials on vulnerability assessments and to co-ordinate different government departments’ programmes on HIV and nutrition. A relationship with the Geography Department at the University of Witwatersrand has provided opportunities to work with food security specialists including part-funding of a PhD candidate. Recently funded projects include one into the role of the environment as a safety net in the context of high HIV prevalence; another on migration, food security and HIV in an urban-rural links project in Johannesburg; and a further study into the health requirements of a comprehensive strategy for dealing with migrants in Johannesburg after the recent criminal and xenophobic violence.

RENEWAL has also made itself available to the Food Security Directorate at the National Department of Agriculture and the Food Insecurity and Vulnerability Information Management System (FIVIMS). Engagement with the Department of Agriculture has been a long stop-start process, however, as a result of the latter’s own complex, internal politics that is symptomatic of the heavily politicised HIV/AIDS debate during the presidency of Thabo Mbeki. RENEWAL has stepped carefully, particularly when working with Directorates in the Departments of Agriculture and Health, whose personnel were often limited in what they could say or do.

### The extractive nature of research

A common criticism of researchers generally is that they remove information from communities to feed into research reports and policy dialogues that never return to the local level. Through the leadership of HEARD, RENEWAL has demonstrated how a continuous feedback process back to respondents not only contributes back to community and household level development in AmaJuba District in KwaZulu-Natal but also validates and strengthens the research findings and its relevance.

RENEWAL encourages on-going critical commentary and interpretation of research findings as they emerge by different parties, and engagement of researchers with that process, as a means to strengthen the validity of final analyses and to ensure utilisation of them. However, other researchers do not always acknowledge this rationale within RENEWAL’s facilitation of various modes of communication, including training workshops. RENEWAL has found on occasion that its workshops and training courses have been viewed as means for researchers to write more creatively yet, still, to disseminate their findings when they have completed analyses and to avoid engaging with the political facets of their work.

These challenges are often offset by successes that demonstrate how the slow task of building trust and strengthening relationships can result in a positive shift in mindsets. For instance, RENEWAL was approached to support civil society organisations and the government in their understanding and conceptualisation of food and nutrition security following revitalisation of the South African National AIDS Framework (SANAF) in 2007. Similarly, in 2008, following persistent engagement with the Department of Health, RENEWAL was invited to help facilitate a mini-conference organised by senior officials who wanted to stimulate wider discussion within government about the HIV-Hunger nexus. Likewise, ongoing discussion with representatives of the Integrated Food Security Strategy (IFSS), which part monitors food insecurity in the country, has led to consideration of a training workshop on HIV and food insecurity indicators.

### Strengthening capacity

RENEWAL has made a general distinction between training on scientific practice and training on thematic knowledge. This is one way of indicating that there are steps between conducting research and using the results. Material has been developed by IFPRI in collaboration with the RENEWAL team and training modules have been made available through the Africa Centre for Food Security.

Initiatives include online courses on proposal writing and scientific writing for publications. Members of the Advisory Panel and researchers linked to the network attended an intensive training course which contained modules on research methodologies and building and analysing networks, as well as opportunities to exchange work experiences and advice from IFPRI researchers, and sessions to develop communication techniques, specifically writing, and skills such as leadership and management of projects. In 2009, the programme began to offer, at the request of the country networks, training workshops on mapping the policy environment and writing policy briefs.

## Discussion

The paper has discussed how and why ‘boundary work’ may be an important concept and approach in attempts to facilitate evidence-based policies and programmes. A network or set of networks is one basis on which researchers and policy makers can influence each other and, hence, be a source of evidence-based interventions. Nonetheless the RENEWAL experience has highlighted out four main operational challenges.

The first challenge is the nature of politics and political engagement. RENEWAL in South Africa has deliberately focused on supporting government officials who have been interested in evidence to inform programmes. However, these officials have been constrained by the peculiar politics of HIV/AIDS in the country that prevented open dialogue within the government. In addition, labyrinthine departmental politics has, in some cases, stymied planned meetings between officials and RENEWAL’s network of researchers. In hindsight, the NAP could have been pushed to promote the RENEWAL agenda more frequently in their own circles. However, real constraints influenced this in terms of focusing on sustaining the relationship and seeing this as a necessary focus until such time that the NAP broached discussion on a more active role.

The second challenge is the maintenance of the integrity of the interactive research agenda. This involves adherence to principles of science whilst maintaining close relationships with those with political authority and also ensuring accountability to the communities within which the research is conducted. Key premises for juggling these matters are, to think and act constructively in terms of the “powerlessness” of the researcher and to “reach-in” to what individuals and organisations share rather than to focus on differences.

The third challenge is selecting and nurturing ‘champions’ in government departments and in scientific organisations. Senior officials regularly move to new posts in and beyond government; hence, selecting and nurturing ‘champions’ is an ongoing process. Researchers have a ‘natural’ reticence against the communication and use of their research reports and publications in different forms and ways. Some struggle with the idea, let alone the practice, of engaging in critical commentary and interpretation of research findings with different ‘non-science’ parties, to strengthen the validity of final analyses and to ensure utilisation of them. RENEWAL’s response has been to support training on various forms of communications and exploration of the interactive research agenda by postgraduate students involved in RENEWAL projects. As Kees Swaans wrote in his doctoral thesis [29]:

“I especially welcome the [RENEWAL] program for its willingness to move beyond understanding the relation between HIV/AIDS and agriculture, and that it actually stimulates new types of inter-disciplinarity and action-orientated research to gain more insight in how to respond to the HIV/AIDS pandemic”

The fourth challenge is the seemingly interminably slow process of influencing policy/decision-makers which requires a long-term perspective and ‘knowing’ that this will bear fruit in due course. A simple lesson learned is that a functioning boundary organisation needs to be persistent; more precisely, to adopt an informed, supportive, flexible and adaptive approach. The gradual strengthening of networks allows trust to be built while bringing in and securing diverse representatives is a key source of legitimacy and, hence, influence.

These considerations have led RENEWAL to pay due attention to the communication of research. The imperative of IFPRI and HEARD has been to secure the credibility and legitimacy of RENEWAL on the basis of the quality of the evidence gained from scientific research. Enabling ongoing critical commentary of research findings by different parties has involved learning to package and present evidence in different ways. New information and communication technologies are increasingly vital for that purpose. Nonetheless, developing more pathways within the network and from the NAP into government, as well as combining opportunities for thematic debates in training courses have been equally important. In sum, the integration of research and communications is a critical endeavour for a boundary organisation.

## Conclusion

Reflecting on the use of the concept of a boundary organisation in framing the experience of RENEWAL in South Africa, a number of conclusions can be reached.

A key argument has been that policy processes are rarely logical, particularly in a terrain as fraught as that of HIV and food security in South Africa. In order to engage the diversity of actors involved, RENEWAL set up a “safe space” for mid-level civil servants, civil society and research and academia to engage these issues – and ultimately to build confidence and expertise to shift policy as it became politically feasible. This reflected a key lesson that acting as a boundary organisation involves adopting an attitude of becoming a “policy entrepreneur” with a long-term view.

Building on this, another important lesson was that RENEWAL played the role of a boundary organisation through a networking approach. As emphasised throughout the paper, boundary organisations involve the participation of actors from both sides of the boundary, as well as individuals and organisations who play a mediating role with distinct lines of accountability to each. This was never formalised largely as a result of wanting to retain an element of informal exchange and support, which in retrospect may have been a limiting factor when the political opportunities to shift policy emerged. Thus a boundary organisation must be alert to changing its emphasis particularly when the policy dialogue shifts.

RENEWAL has not claimed to have directly shifted policy. It has rather seen itself as contributing to broader processes of policy change that have involved building the evidence available to policy makers, strengthening the capacity of certain individuals and groups to use that evidence, to consolidate relationships that could drive a policy agenda and to provide a background source of support available to decision makers at various levels. The most pertinent is the support provided to civil society organisations and government in their understanding and conceptualisation of food and nutrition security through the South African National AIDS Framework. Similarly, the invitation to help facilitate a mini-conference organised by the Department of Health to stimulate wider discussion within government about the HIV-Hunger nexus. These, and others, demonstrate how RENEWAL as a boundary organisation has been able to play different supporting roles in policy-making processes.

RENEWAL has made a contribution through research projects informed by the networking approach to build bridges between actors, which have fed into broader processes that have led to changes. Examples reveal that researchers have to build relationships with actors that have the capacity to make changes, whether they be civil society or government, which reveals the instrumental value of research and the credibility of the research teams. Thus there is a strong argument for groups hoping to influence policy making to utilise the concept of a boundary organisation, particularly as it helps break down the linear approach to such processes, and helps the understanding of how “messy” processes can be engaged through networks and alliances.

## Competing interests

This article critically reflects on a research project in which the authors have been involved."

## Authors’ contributions

The authors contributed equally to the manuscript

## Authors’ information

Scott Drimie, PhD, is the Regional Co-ordinator of the Regional Network on AIDS, Livelihoods and Food security (RENEWAL), a programme of the International Food Policy research Institute (IFPRI).

Tim Quinlan, PhD is the Research Director of the Health Economics and HIV/AIDS Research Division (HEARD) at the University of KwaZulu-Natal.
